# α‑Gal Causes Tick-Related Meat Allergies—It Could Also Be a Therapeutic

**DOI:** 10.1021/acscentsci.5c02233

**Published:** 2025-12-10

**Authors:** Marta Zaraska

## Abstract

As people with α-gal syndrome seek to boost awareness, researchers are looking to harness the molecule for new therapies.

## Graphical abstract

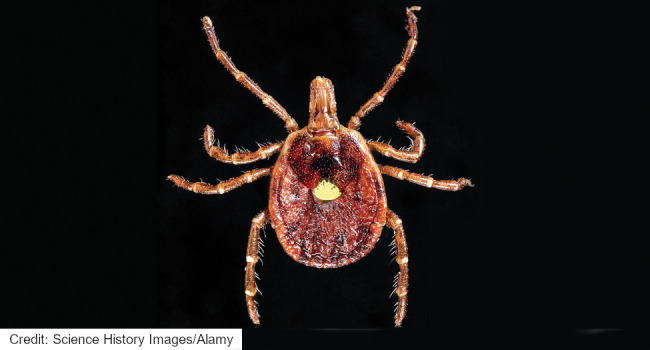

The first time Sage Scott felt sick on a taco night she thought it was food poisoning. But the story kept repeating itself over the coming days: first it was a hot dog, then a steak—and no one else who ate the food seemed to suffer.

“I basically crawled from the dinner table to bed,” she says. Scott’s family physician diagnosed her with an allergy to galactose-α-1,3-galactose, or α-gal, an emerging condition triggered by tick bites and often referred to as a meat allergy. Scott, an outdoor photographer, was not exactly surprised. There are ticks everywhere where she lives in Missouri, and the condition is fairly well-known in the area.

What Scott’s doctor got wrong at first, however, was the care plan: she told Scott to just avoid red meat. Although her extreme gastrointestinal reactions on Taco Tuesdays stopped, Scott still suffered rashes and joint pain.

**Figure:**
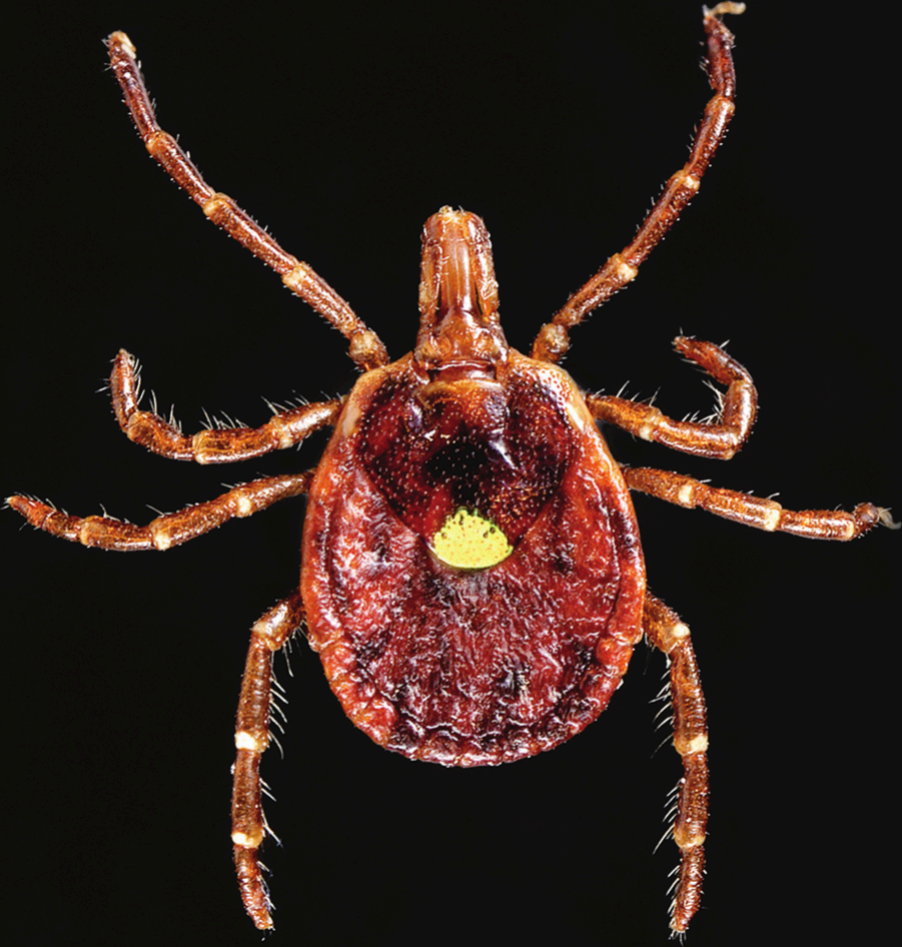
Bites from the lone star tick can cause allergies to galactose-α-1,3-galactose, a sugar common in animal products such as red meat. Credit: Science History Images/Alamy.

It took several months for Scott to understand the full scope of her condition. α-Gal is a disaccharide present in not only red meat but a slew of animal-derived products, including the gelatin capsules that enclosed Scott’s dietary supplements. That meant Scott essentially had a drug allergy, too.

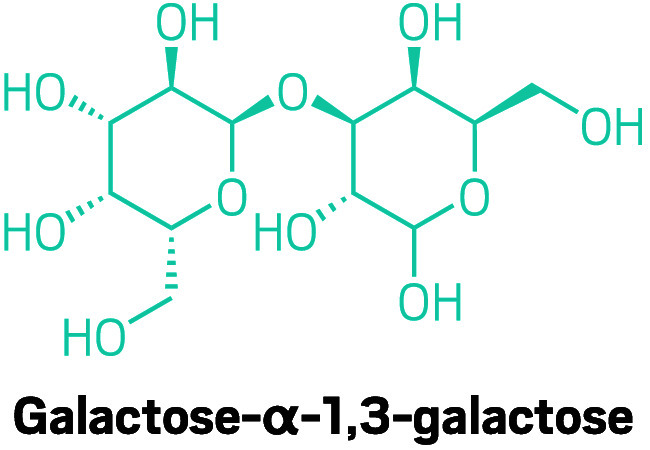



α-Gal is naturally present in many mammal-derived pharmaceuticals and medical devices, including collagen sutures, some vaccines, anesthetics, and IV solutions. Even bandages can cause problems if they’re made with mammal-derived adhesives. The allergic reaction Scott experiences, also known as α-gal syndrome, has been described all over the globe, including in North America, Australia, Europe, and Africa. According to the US Centers for Disease Control and Prevention (CDC), there are 450,000 cases in the US alone, triggered mostly by the bites of the lone star tick, whose range is expanding because of climate change. It is an unusual allergy, since the symptoms are often delayed by 3–5 h. The reaction manifests as abdominal pain and vomiting in most people, but others experience rashes, breathing problems, and, like Scott, joint pain.

Scott now gets her medication from a compound pharmacy that puts the active ingredients in a plant-based capsule. In the US, emerging platforms and advocacy groups are pushing for more transparent drug labeling and are helping people find α-gal-free drug alternatives. But such alternatives are not always available, and misinformation abounds.

Simultaneously, scientists are discovering that α-gal could have some redeeming qualities. Because of the way the sugar can trigger the body’s immune system, researchers are trying to harness it for a variety of therapies. Understanding the molecule’s unusual features could lead to better vaccines, faster ways to heal wounds, and even new cancer treatments, as evidenced by clinical trial results published this year.

## α-Gal is (almost) everywhere

In the 1980s, Uri Galili, then at the University of California Medical Center and now retired from Rush University Medical Center, discovered that human blood contains “large amounts” of an antibody that binds best to α-gal. It was a carbohydrate antigen that hadn’t got much attention and had not yet earned its shortened moniker.

At the time, Galili lived near the San Francisco Zoo and had easy access to animal blood samples. In his analyses, he noticed a pattern: although Old World monkeys, apes, and humans did not synthesize the carbohydrate, all other mammals did.

Galili began hypothesizing and figured that perhaps an epidemic 20–30 million years ago killed off Old World monkeys and apes whose bodies made α-gal. Since many viruses and bacteria carry α-gal on their surface, individuals who did not produce the molecule—and thus made antibodies against it—survived. Today, research suggests that antibodies for α-gal help us fight Chagas disease, leishmaniasis, and tuberculosis.

Although these antibodies benefit the vast majority of us, some people develop an allergy to the molecule after being sensitized by a tick bite. α-Gal “is an important component of tick cement, which is what a tick produces in order to stay attached to the host,” says José de la Fuente, a molecular biologist at Oklahoma State University and Instituto de Investigación en Recursos Cinegéticos. When a tick bites a human, it may trigger production of immunoglobulin E (IgE), an antibody that can cause allergies. In contrast, IgG and IgM are the key infection-fighting antibodies.

In meat, large glycoproteins are studded with α-gal. These sprawling biomolecules are “very resistant to digestion,” says Christiane Hilger, a molecular biologist at the Luxembourg Institute of Health. “It takes hours for allergic reactions to occur.” Other food allergies tend to come on far more quickly.

A much faster response may happen when α-gal is delivered directly into the blood. α-Gal syndrome was first reported in 2008, when severe allergic reactions to an intravenous cancer drug—the monoclonal antibody cetuximab—were linked to the α-gal it contained.

Some patients infused with cetuximab experienced anaphylactic shock within minutes. Since cases were more common in areas with a high population of lone star ticks, such as Tennessee and Arkansas, the arachnids were suggested as a potential culprit.

Another drug of concern is heparin, a polysaccharide that is derived from pig intestines and cow lungs and is used as an anticoagulant. “Heparin becomes an issue in part because it’s so commonly used in the hospital,” says Jeff Wilson, a clinical immunologist at the University of Virginia.

One typical case is heart surgery. In one of Wilson’s studies, almost a quarter of people sensitized to α-gal had a severe allergic reaction to heparin during those operations—though the sample was very small.

Antivenoms may trigger a reaction, too. Most are made from horse or sheep blood and may contain α-gal. In 2016, Hilger and her colleagues did a cell experiment confirming a link between antivenoms and allergic reactions to α-gal.

On the US market, tens of thousands of drugs contain α-gal, estimates Sachin A. Shah, a pharmacist and cofounder of Pill Clarity and Pill Clarity Foundation. The former offers transparency and safer alternatives to products that contain α-gal, and the latter is an advocacy and education nonprofit.

Gelatin is also used as a tablet binder and a plasma expander to increase blood volume in patients with blood loss. Clinicians have already reported anaphylaxis cases as a result of such products.

Pill Clarity receives about 1,000 inquiries a month from people wanting to know which drugs are α-gal-free. “I’m getting patients calling me from the ER saying, ‘Hey, what do I do? These guys are not taking me seriously,’” Shah says.

When Sage Scott had to go to the ER for a bad cut, her doctor didn’t know about α-gal. “I was the one who had to google which medicines are OK,” she says. In a survey conducted by the CDC, 42% of US health-care providers had never heard of the α-gal syndrome. “We clearly have a lot more to do on the education side,” says Scott Commins, an immunologist at the University of North Carolina at Chapel Hill.

Drugmakers are currently not required to disclose the presence of animal-derived ingredients in their products, even though “labeling would probably be the quickest, easiest solution,” Commins says.

According to Sharon Forsyth, who has α-gal syndrome and cofounded the advocacy nonprofit Alpha-gal Alliance Action Fund, clear drug labeling is “desperately needed.” The action fund has endorsed a bill recently introduced in the US Congress that would codify α-gal as a major food allergen and require it to be labeled on packaged foods alongside soy and peanuts. This could also be the first step toward the labeling of medical products, Forsyth says.

Yet for now, even some drug manufacturers can’t immediately say what’s in their products. A 2023 study in the *Journal of Contemporary Pharmacy Practice* conducted by Pill Clarity (then called VeganMed) found that 40% of pharmaceutical companies’ medical information departments couldn’t provide accurate information about animal-derived ingredients (PDF) in their medications.

What’s more, Commins says, the drugs’ α-gal content may vary between manufacturers and batches. For Wilson, a simple test to measure a pharmaceutical’s α-gal content would be great, but nothing like this yet exists. “α-Gal is hard to measure,” he says. The sugar can be connected to a wide variety of mammal proteins and lipids. Lipids in particular present a challenge because of their low solubility in water. “Put it all together and it makes for a real technical challenge,” Wilson says. But he adds that he “would love to team up with a chemist” to design such a test.

Many drugs that contain α-gal do have safe alternatives; these are either already available on the market or can be custom-made by a compounding pharmacy. “From a pharmaceutical standpoint, you could certainly just choose to use plant-based gelatin equivalent or magnesium stearate,” Commins says. Around 80% of the 10,000-plus unique medication inquiries to Pill Clarity this year had an animal-free alternative, according to Shah.

But not much is being done for those without a viable substitute. “I don’t know a single big pharma company that has a forward-thinking plan saying, ‘We’re going to work around this to ensure patient safety,’” Shah says.

## α-Gal has another side

While α-gal challenges doctors who treat tick-sensitized patients, other researchers, such as Galili and de la Fuente, also see the molecule’s potential to save lives. α-Gal is a double-edged sword: in people with α-gal syndrome it stirs up IgE antibodies associated with allergies, but the molecule can also trigger production of IgM and IgG antibodies which help fight infections.

“You can get protection without the allergy, or you can get the allergy and then also protection,” de la Fuente says. He and his colleagues evaluated various α-gal-producing probiotic bacteria they isolated from traditional fermented foods such as pulque, agave wine, and a sour yogurt called kindirmo. Their experiments showed that such bacteria can act as probiotics and stimulate protective antibodies in mice.

In other work, de la Fuente and colleagues found that vaccination with α-gal in zebra fish triggered a strong immune response against tuberculosis. Since α-gal is found on many pathogens, not just tuberculosis bacteria, de la Fuente says that these results suggest the possibility of a future “pan-vaccine” capable of simultaneously controlling several infectious diseases.

Galili and his colleagues have been experimenting with mice engineered to lack α-gal, which are useful models for human immune responses. He created α-gal nanoparticles made from rabbit lipids that he and his team administered in various injured tissues in the mice.

After injection, skin wounds, burns, and injured heart tissue regenerated and healed free of scars. The nanoparticle treatment could even trigger severed nerve fibers in crushed spinal cords to reconnect.

“Once the nanoparticles are administered to the injury, they bind the anti-Gal antibody, and this interaction initiates the healing cascade,” Galili says. Specific types of white blood cells that promote tissue regeneration start migrating to the injury site and “orchestrate the regenerative processes,” he adds.

**Figure:**
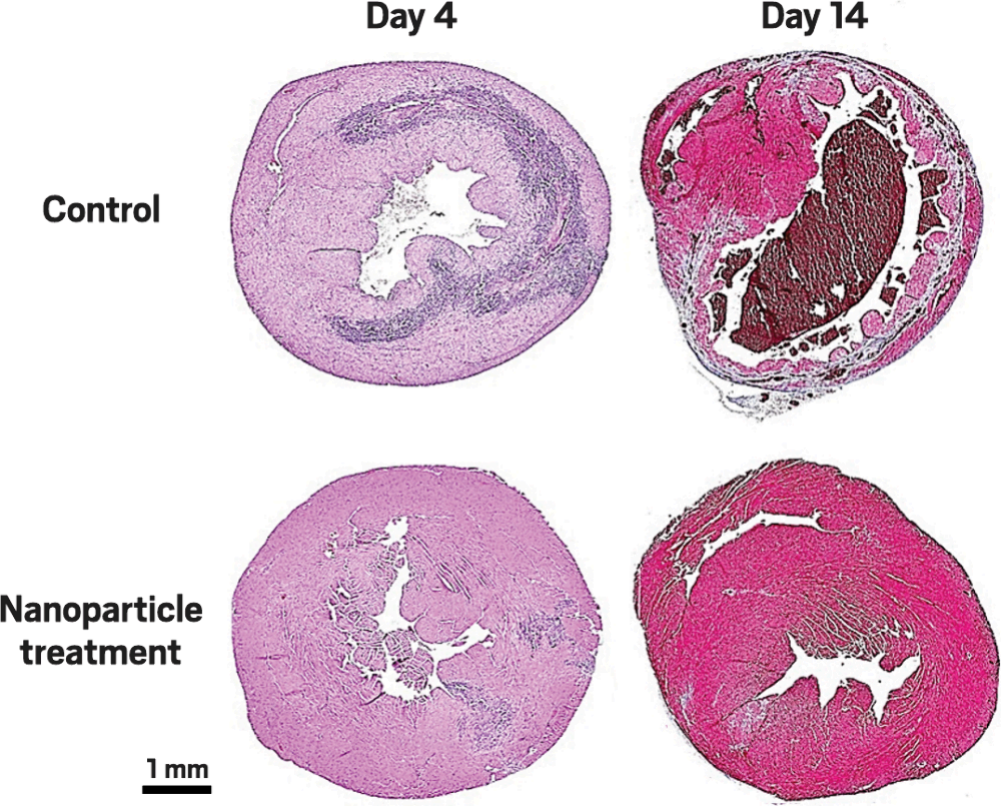
**Mend a broken heart**. Two weeks after a heart attack, a mouse heart tends to develop scars (top right, brown), but treatment with nanoparticles containing galactose-α-1,3-galactose allowed tissue to regenerate without scars. Credit: *Nanomaterials*/C&EN.

**Figure:**
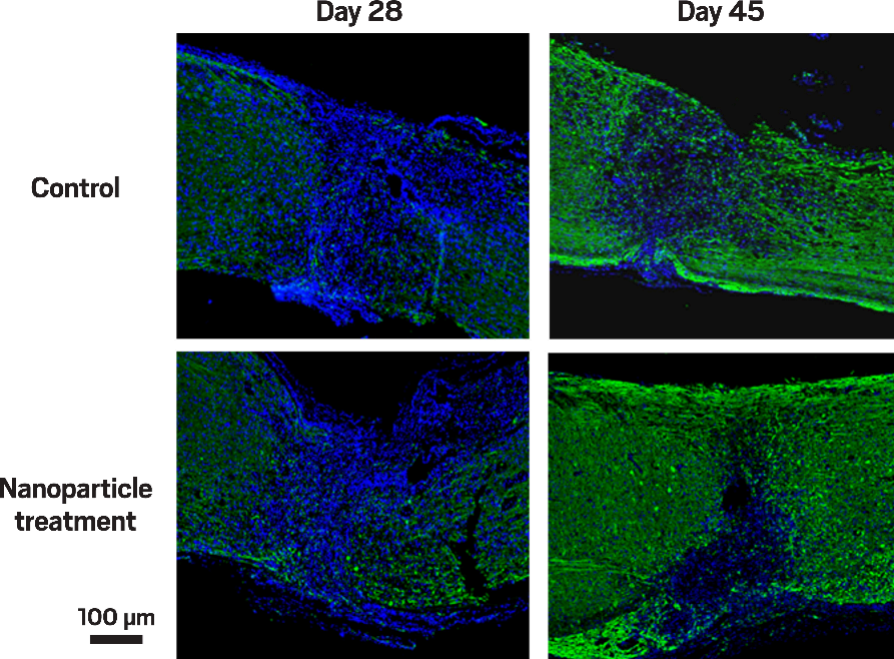
**Neural regeneration**. Nerve fibers (green) reconnected more effectively in mice’s spinal cords treated with galactose-α-1,3-galactose. Credit: *Nanomaterials*/C&EN.

Then there are cancer therapies. In a small 2025 clinical trial that Galili calls “very promising,” a team of researchers in China modified a virus to deliver a pig gene into human tumor cells, thus inciting the cells to produce α-gal. The molecules then acted like a bull’s-eye for patients’ IgM and IgG antibodies, which attacked the tumors. In 20 people with advanced disease, including liver and lung cancers, the vast majority of tumors shrank or even disappeared, with few side effects.

However promising, α-gal therapies might be problematic for people sensitized to the molecule by ticks. The good news is that the syndrome tends to wane with time. Research shows that blood levels of IgE antibodies against α-gal gradually decrease since the most recent bite over a few years, Commins says. If you can “avoid additional tick bites for 3–5 years, which admittedly can be very difficult to do,” your IgE antibody levels against α-gal “will often be undetectable,” he says. For those with severe reactions or whose symptoms tend to remain prominent, the anti-IgE monoclonal antibody omalizumab may offer hope.

Sage Scott realizes that as she gets older, she will probably be taking more drugs and will have to be mindful about their contents. For immunologist Wilson, navigating α-gal is a “balancing act.” He sees many patients who have been online reading and chatting and who end up avoiding everything under the sun containing α-gal.

“I don’t want people to go down this rabbit hole,” Wilson says. He adds that the public should be trying to gather more knowledge without getting overly worried, which can happen easily if you go online. And we certainly do need knowledge: information about α-gal content in drugs and about safe replacements, as well as data on how scientists could use α-gal to develop new treatments.

In the meantime, though, “stay away from more tick bites,” Wilson says.

*Marta Zaraska is a freelance contributor to* Chemical & Engineering News, *the independent news outlet of the American Chemical Society.*

